# Toward the Prediction of Multi-Spin State Charges of a Heme Model by Random Forest Regression

**DOI:** 10.3389/fchem.2020.00162

**Published:** 2020-03-31

**Authors:** Wei Zhao, Qing Li, Xian-Hui Huang, Li-Hua Bie, Jun Gao

**Affiliations:** Hubei Key Laboratory of Agricultural Bioinformatics, College of Informatics, Huazhong Agricultural University, Wuhan, China

**Keywords:** spin crossover, heme model, force field, machine learning, ESP charge

## Abstract

The random forest regression (RFR) model was introduced to predict the multiple spin state charges of a heme model, which is important for the molecular dynamic simulation of the spin crossover phenomenon. In this work, a multiple spin state structure data set with 39,368 structures of the simplified heme–oxygen binding model was built from the non-adiabatic dynamic simulation trajectories. The ESP charges of each atom were calculated and used as the real-valued response. The conformational adapted charge model (CAC) of three spin states was constructed by an RFR model using symmetry functions. The results show that our RFR model can effectively predict the on the fly atomic charges with the varying conformations as well as the atomic charge of different spin states in the same conformation, thus achieving the balance of accuracy and efficiency. The average mean absolute error of the predicted charges of each spin state is <0.02 e. The comparison studies on descriptors showed a maximum 0.06 e improvement in prediction of the charge of *Fe*^2+^ by using 11 manually selected structural parameters. We hope that this model can not only provide variable parameters for developing the force field of the multi-spin state but also facilitate automation, thus enabling large-scale simulations of atomistic systems.

## 1. Introduction

Coordinated compounds of transition metal ions can exhibit a switching phenomenon under certain conditions related to changes in temperature, pressure, light, or magnetic field; the central metal ion changes the spin states (the so-called high-spin, HS, and low-spin, LS, configurations), which is the spin transition (ST) or spin crossover (SCO) (Bousseksou et al., 2011; Gutlich et al., 2013). Since Cambi et al. first reported the thermally induced change of spin states in 1931, (Cambi and Szegö, 1931) many more SCO complexes have been synthesized thereafter and have been applied to various domains, including molecular switches, memory elements (Jureschi et al., 2014; Shao et al., 2015), temperature sensors (Gütlich and Goodwin, 2004; Doukov et al., 2011), nanomaterials (Nagl et al., 2008; Hauser, 2013), and so on (Bousseksou et al., 2011; Cong et al., 2018; Yuan et al., 2018; Meyer et al., 2019).

In the switching phenomenon, the change of spin state is accompanied by a switch of electron configurations of the central ions, which often leads to marked changes in the physical and chemical properties of the entire complex (Gütlich and Goodwin, 2004; Habenicht and Prezhdo, 2012; Gutlich et al., 2013). Meanwhile, the reorganization of electrons among atoms and the formation of molecules are complex and multifaceted processes, and their full description is only possible within the boundaries of quantum mechanics (QM) (Bristow et al., 2014; Sanvito, 2019). Density functional theory (DFT) is the most common choice for routine ground-state calculations; however, the number of valence electrons scaled cubically, increasing the computational costs significantly (Engler et al., 2019). It will therefore not be suitable, especially when one needs to sample extended size and time scales.

Molecular dynamics (MD) simulation can handle system sizes of typically 10^7^ atoms and above, and this has been used for decades to explore chemical and biochemical problems at an atomic level (Liu et al., 2017; Riniker, 2018). The classical MD predominantly uses simplified atomistic models called force fields (FFs) to describe the exact ground-state potential energy surface (PES) of a system. The bonded parameters are represented in terms of equilibrium bond distances, bond and dihedral angles, force constants, and rotation barriers; the non-bonded interactions are typically described by atom-centered point charges and Lennard-Jones potential (Ivanov et al., 2015) while disregarding the explicit treatment of electronic polarizability (De et al., 2018; Sahoo and Nair, 2018; Heid et al., 2019). It is not capable of capturing a restricted but essential number of chemical features, including spin crossover, wherein the molecular system is required to “hop” from one PES of the initial spin state onto another of the product state.

In order to better understand the effect of molecular properties on their electronic ground or excited states, the potential parameter set needs to be extended by a multi-spin state in which at least two issues should be taken into account. Firstly, the geometric configuration at energy minima of the excited state is different from that of the ground state in most cases. This issue can be fixed by adjust the parameters in bonding terms. For example, Meyer's Group has modified force constants for bond stretching and bending terms according to DFT calculation for atomistic molecular dynamics simulations of the HS and LS states of the *Fe*^2+^ containing model (Meyer et al., 2019). Secondly, it is well-known that the charge distribution in the excited state is different from the ground state, and it will change with molecular structures; it is important for the force field to provide the charges of two spin states. In this regard, an increasing number of schemes have been proposed in addition to the polarized force field, such as the SSAPs method (Xu et al., 2018).

In recent years, many efforts have been directed to the efficient improvement of force fields. In particular, machine learning combined with molecular simulation has been verified by many groups to be effective to develop force field including inferring charges based on a set of reference molecules (Botu et al., 2016; Chen et al., 2018; Inokuchi et al., 2018; Engler et al., 2019; Hu et al., 2019; Roman et al., 2019; Sanvito, 2019; Unke and Meuwly, 2019; Ye et al., 2019). Among these, the random forest regression (RFR) method has been proven to be feasible for the prediction of atomic charge without expending much effort on parameter tuning or descriptor selection. As a classification and regression tool, the Random Forest algorithm was first introduced by Breiman (2001), inspired by the earlier work of Amit and Geman (1997). It uses bootstrap samples of the training data and random feature selection in tree induction. Each tree in the ensemble produces an output according to the molecular descriptors or properties, and outputs from all trees are aggregated to produce the final prediction by average (Breiman, 2001; Cutler et al., 2011). This procedure can reduce overfitting and offer some unique features, including built-in performance assessment and measures of variable importance (Svetnik, 2003; Klusowski, 2018), which make it suitable for quantitative structure-activity relationship (QSAR) tasks (Svetnik, 2003; D Richard et al., 2007; Statnikov et al., 2008; Genuer et al., 2010). For instance, Rai and Bakken (2013) combined random forest regression with ESP charges from high-level QM calculations to predict the partial atomic charge of H, C, N, O, F, S, and Cl. Building on their work, Bleiziffer et al. (2018) further presented a conformational robust charge extraction scheme DDEC to predict partial charges and achieved accuracy beyond a HF/6-31G* setup. Our group developed a conformational adaptive charges (CAC) model based on atom type symmetry function (ATSF), which was, in turn, based on the RFR method (Wang and Gao, 2020). These machine learning approaches in tandem with quantum mechanics have many merits in developing flexible and adaptive force fields, including low cost, accuracy, and versatility. Yet, they are mainly used to predict charges on the single potential energy surface of the equilibrium configuration of the molecule. The performances of these method on multi-spin state charges remains unreported.

In our previous work (Liu et al., 2017; Du et al., 2018), the spin-forbidden dioxygen binding dynamics in a simplified heme model were investigated by the non-adiabatic trajectory surface-hopping dynamics, and this involved the coupled singlet, triplet, and quintuplet states. The results revealed that there existed dominant long-lived, kinetically meta-stable states during the dynamics trajectories, and each meta-stable pattern showed a distinct partial charge population. Based on this geometric dependence of the partial charge population on the excited state, we proposed to extend the conformation adapted charge (CAC) model and RFR method to the multi-spin state charges of the heme model. The fixed-point charge in the traditional force field can be modified according to the conformation on the fly, and thus the key to the multi-spin state is transformed into the change of charge in the multi-spin state. We hope that this model can not only provide variable parameters for constructing the force field of the multi-spin state but also facilitate automation, thus enabling large-scale simulations of atomistic systems.

## 2. Materials and Methods

**Figure 1:**
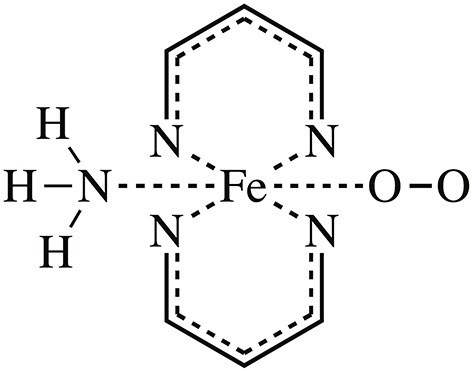
The molecular model of this work. The simplified heme model $Fe^{2+}{\left(C_{3}N_{2}\right)}_{2}NH_{3}$ complex with *O*_2_ binding was adopted.

**Figure 2:**
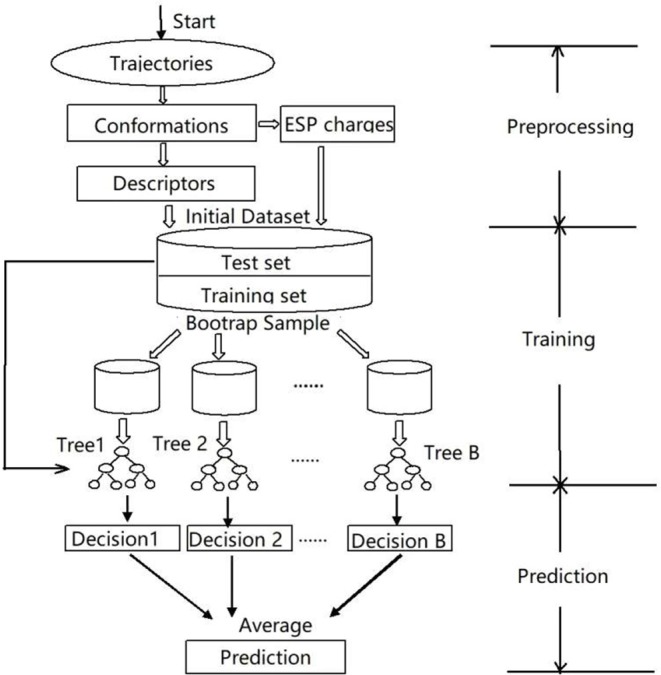
The work flow of construction data set and prediction process of the RFR model.

In this work, we targeted the simplified heme model (see Figure 1), introduced a random forest regression (RFR) algorithm using Behler-Parrinello symmetry functions as descriptors (Behler et al., 2007; Hagai et al., 2010; Behler, 2011a), performed model training by fitting ESP charges of different spin states, and achieved high-quality predictions. The key steps of the workflow are shown in Figure 2. The samples sufficient molecular conformations were obtained from *ab initio* dynamic trajectories of previous work (Du et al., 2018), which covered a wide range of conformations related to the spin crossover. Different descriptors were then extracted, and the ESP charges of three spin states of each atom in each conformation were calculated using the density function theory method, and together these constitute the initial dataset. After this preprocessing was completed, half of the data were selected randomly as the training set to build the RFR model, and the remaining half of the data were used to test the model's ability to reproduce the atomic partial charge under different spin states and thereby to analyze and assess the performance of the model.

### 2.1. Data Set Preparing

A total of 33 stable trajectories of open-shell singlet state were selected from a non-adiabatic trajectory surface-hopping dynamics simulation from our previous work. The B3LYP/6-31G* level of the method (Reiher et al., 2001; Salomon et al., 2002) was used to calculate the ESP atomic charge of each structure in the singlet, triplet, and quintuplet state. We finally achieved 39,368 converged structures owing to the convergence of the calculation. The data preparation was time consuming. By and large, it took 2 weeks to complete all the calculations of the 39,368 structures for each spin state with four computer nodes; each node had dual Intel 2683v3 CPUs. All the electronic structure calculations were implemented with a Gaussian 16 package (Frisch et al., 2016), and the detail charge distribution of each atom in the different spin states were analyzed and shown in the section 3.

### 2.2. Random Forest Regression Model Training

The raw dataset was preprocessed firstly to extract appropriate features, such as the descriptors of structures and input of model. Specifically, each RFR model was constructed separately under certain spin states for each atom according to the flow shown in Figure 2. Since there were 14 atoms in the simplified heme model, 14 independent RFR models were constructed by training for each spin state. There were 42 models in total.

Let *D* = {(*x*_1_, *y*_1_), ⋯⋯, (*x*_*N*_, *y*_*N*_)} denote the training data, with *N* = 39368/2, $x_{i}={\left(x_{i,1},\cdots \cdots x_{i,p}\right)}^{T}$representing the information relative to atom *i* in each structure described with *p* features, and *y*_*i*_ denoting the ESP charge. During the training process, for each decision tree in the forest, a bootstrap sample *D*_*j*_ from the training data of *N* molecules was drawn first. Starting with all observations (*x*_1_, *y*_1_)⋯⋯(*x*_*N*_, *y*_*N*_), of *D*_*j*_ at each node, *m* predictors were selected at random from the p predictors (m < p), and the node was split into two descendant nodes using the best split among the remaining predictors. This process was repeated until no further splits ere possible to grow a tree, and the steps were repeated again until all the trees were grown.

Although Random Forests can obtain good results using the default parameters in most cases, appropriate parameters can further improve the accuracy for particular situations. There is only one parameter to which random forests is somewhat sensitive—*m*. This denotes the number of randomly selected predictor variables at each node. The default value of *m* is often set by *p*/3. In the RFR model, combined with symmetry functions, different values of *m* were tested, and, finally, *m* = 5 was determined by comparing the Pearson correlation coefficient (r) between the predicted charges and the ESP charges of *Fe*^2+^. Another parameter, *B*, which represents the number of trees in the forest, can be chosen to be as large as desired; Breiman (2001) showed the generalization error for random forests converges almost surely to a limit as B increases. Here, B was set as 200.

When the training is completed, the prediction charge of a given atom i in a new geometry structure will be given by the average prediction of all individual trees. Thus, the predicted charge is assigned as Equation (1):

(1) $$\begin{array}{l} \bar{q_{i}}=\frac{\sum_{j=1}^{B}T_{j}\left(x_{i}\right)}{B} \end{array}$$

The standard deviation of the predicted charge for atom i by the tree T is defined as Equation 2:

(2) $$\begin{array}{l} \sigma_{i}=\sqrt{\frac{\sum_{j}^{B}{\left(q_{i}\left(T_{j}\right)-\bar{q_{i}}\right)}^{2}}{B}} \end{array}$$

where *q*_*i*_(*T*_*j*_) is the partial charge predicted by tree *T*_*j*_. The RFR algorithm was implemented using the scikit-learn module in Python.

### 2.3. Descriptor Selection

To encode the physical features and the mandatory symmetries of the problem, many descriptors have been introduced (Imbalzano et al., 2018). For example, Huan et al. (2017) utilized a d-dimensional vector *V*_*i*, α_, representing the atomic environment of atom *i* viewed along the Cartesian α direction (Huan et al., 2017). Heid et al. (2019) used the type of each atom and its connectivity as the input for the neural network. Schutt et al. (2017) introduced a vector of nuclear charges and a matrix of atomic distances to describe the molecular structures. In addition, molecules can be represented as Coulomb matrices (Rupp et al., 2012; Lilienfeld, 2015), scattering transforms (Hansen et al., 2015), bags of bonds (Bartók et al., 2010; Bartók et al., 2013), and so on. Among these various descriptors, atomic-based symmetric function, which was first proposed by Behler et al. (2007), has been widely used in machine learning (Behler et al., 2007; Behler, 2011a,b). Here, we adopted this method to describe the molecular structure.

Atom-based symmetric functions describe the chemical environment of atom *i* in terms of radial and angular terms. Therefore, each atom's Cartesian coordinate *R*_*i*_ = (*x*_*i*_, *y*_*i*_, *z*_*i*_) needs to be converted into the so-called symmetric function form of Equation (3):

(3) $$\begin{array}{l} R_{i}=\{G_{i}^{angular},G_{i}^{radial}\}\\ \;=\{G_{i,\;E_{1}}^{radial},\cdots ,\;G_{i,E_{n}}^{radial},G_{i,E_{1},E_{1}}^{angular},\cdots ,\\ \;G_{i,E_{1},E_{n}}^{angular},G_{i,E_{2},E_{2}}^{angular}\cdots ,G_{i,E_{2},E_{n}}^{angular},\cdots ,G_{i,E_{n},E_{n}}^{angular}\} \end{array}$$

where $G_{i,\;E_{1}}^{radial}$ represents the total contribution of the distance between all the surrounding atoms, and atom *i*, and $G_{i,E_{i},E_{j}}^{angular}$ represents the angular relationship between any two surrounding atoms and itself. All atoms are distinguished according to their element *E*_*i*_, and the set of symmetric functions of two atoms belonging to the same element are thus the same.

In this study, Equation (4) was used to describe the distance component of each atom, where *R*_*ij*_ represents the distance between atom *i* and *j*. The cutoff function *f*_*c*_(*R*_*ij*_) was introduced in Equation 5 because the atoms in the molecular dynamic simulation may enter or leave the cutoff distance, which can lead to the number of neighbor atoms to be variable. Here, *R*_*c*_ was thus set to 99 Å to include all the atoms, and *R*_*s*_ and η were both set to 1.0.

(4) $$\begin{array}{l} G_{i,J}^{radial}=\overset{j\;in\;J}{\underset{j\neq i}{\sum }}e^{{-\eta \left(R_{ij}-R_{s}\right)}^{2}}f_{c}\left(R_{ij}\right) \end{array}$$

(5) $$f_{c}\left(R_{ij}\right)=\{\begin{array}{ll} 0.5\times \left[cos\left(\frac{\pi R_{ij}}{R_{c}}\right)+1\right] & forR_{ij}\leq R_{c}\\ 0 & forR_{ij}\geq R_{c} \end{array}$$

Equation (6) is the angular component, which defines the angular distribution centered on each reference atom; here, λ = 1.0, ζ = 1.0.

(6) $$\begin{array}{l} G_{i,j,k}^{angular}=2^{1-\zeta }\overset{j\in J\&k\in K}{\underset{j,k\neq i}{\sum }}{\left(1+{\lambda coa\theta }_{ijk}\right)}^{\zeta }\\ \;\times e^{-\eta \left(R_{ij}^{2}+R_{ik}^{2}+R_{jk}^{2}\right)}f_{c}\left(R_{ij}\right)f_{c}\left(R_{ik}\right)f_{c}\left(R_{jk}\right) \end{array}$$

Therefore, through coordinate transformation, the symmetric functions for each atom can be obtained and combined with the ESP charge to finally form the training set as the input of model.

Meanwhile, in order to compare the effect of descriptor selection on prediction performance, 11 structural parameters were manually selected and used as descriptors to train the model. Specifically, the 11 parameters included eight distance values (Fe-N1, Fe-N2, Fe-N3, Fe-N4, Fe-N11, Fe-O12, Fe-O13, and O12-O13), one angle value (Fe-O12-O13), and two dihedral angles (N2-Fe-N1-C10 and N1-Fe-N2-C5). According to our chemical perception, these 11 parameters reflect the features of molecule structure, so they can well-describe different conformations.

## 3. Results and Discussion

### 3.1. Charge Distribution of Multi-Spin State in the Initial Data Set

**Figure 3:**
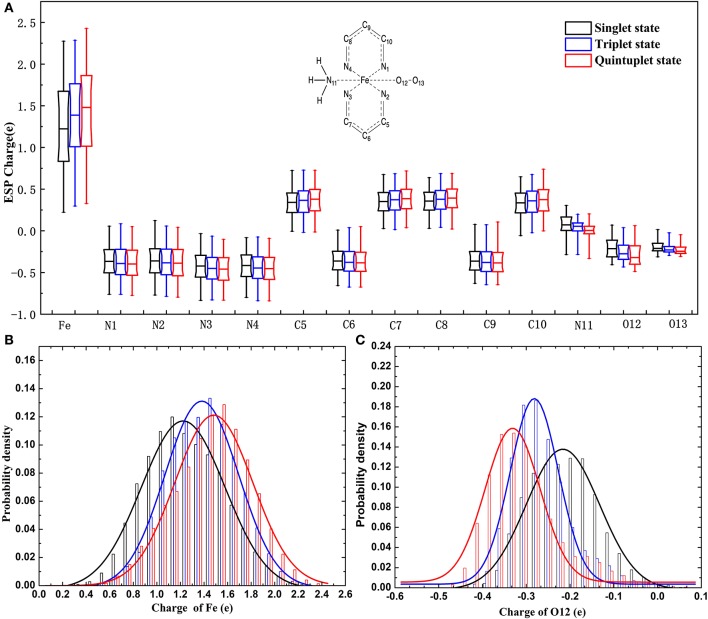
Charge distribution of multi-spin state for 14 atoms of the model in the data set. **(A)** Is boxplot representation of charge distribution of 14 atoms in the heme system in different spin states. **(B)** Is probability density of charge distribution of *Fe*^2+^. **(C)** Is the probability density of the charge distribution of the O12 atom.

**Figure 4:**
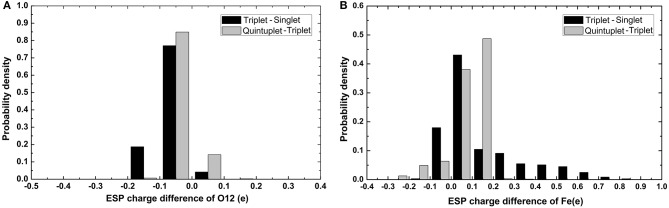
Histogram of charge differences between two spin states of selected atoms. **(A)** Is O12 atom and **(B)** is *Fe*^2+^. The black color code is the charge difference of singlet and triplet and gray color code is charge difference of quintuplet and triplet state.

It can be seen in Figure 3 that most variations range from 0.5 to 0.7e; the fluctuation of *Fe*^2+^ was the most significant, as it was close to 2e. The variation of O12 was larger than that of O13. It can also be found that there was a slight tendency for the mean value of N to decrease and the mean value of C to increase. For Fe and the coordinating O12 and O13, the difference among the mean values under different spin states was relatively more significant. Specifically, the atomic charge of *Fe*^2+^ in the singlet state was distributed around 1.2 and 1.5e in quintuplet. Further analysis of the charge distribution of different spin states showed that the triplet charge of *Fe*^2+^ in most structures was greater than the singlet charge (Δ31 > 0, see Figure 4), with the difference being at the highest probability concentrated at 0.1e, while, for the quintuplet and triplet spin state, the difference reached 0.2e. The results confirmed that different spin states in the same structure had distinct charge distributions.

Additionally, it should be noted that the atomic charge of each atom fluctuated within a certain range, among which *Fe*^2+^ fluctuated the most. Just taking the singlet state as an example, the variation ranged from 0.3 to 2.2e, which implied that the charge distribution of a certain atom in a specific spin state was conformation dependent.

### 3.2. Charge Prediction of RFR Model With Symmetric Functions

In order to better distinguish between different molecular structures, the atom-based symmetry functions were used to convert atomic coordinates into a series of function values, which embed the atoms in their neighborhood depending on the element type (Schutt et al., 2017). It is an efficient way to consider the chemical environments that the invariances, such as translation, rotation, and permutation, can be guaranteed to be exploited by. By doing so, the RFR model combined with symmetry functions and ESP charge was constructed.

As mentioned above, although complex parameter tuning is not required in the RFR model, it is sensitive to the number of descriptors. To this end, we tested and compared the predicted charge of *Fe*^2+^ at different values of *m* (i.e., the number of features selected from p descriptors at random; here *p* = 19) and then calculated the correlation between the predictions and the ESP charges. The results are shown in Table 1. It can be seen from Table 1 that, when *m* = 5, the correlation between the predicted value and the fitted value is the largest (0.9784), which indicated that prediction gave the best performance. The parameter *m* was consequently set to five in the subsequent analysis.

**Table 1:** Tests on the number of features selected in the RFR model.

***m***	**Pearson correlation coefficient**
5	0.9784
0.2	0.9750
log2	0.9771
$\sqrt{p}$	0.9771
19	0.8729

*When using the symmetric function method, each molecular structure is described by 19 features (p = 19), and m is the max features randomly selected from it to fit a tree*.

To assess the prediction performance of the charge models, the mean absolute error (MAE) was calculated for each atom in the three spin states, and the standard deviation of the error was given as well (Table 2). According to Table 2, the MAEs of the predicted charges in three spin states are all within 0.015e for all the spin states. There was no obvious difference between two states. For each state, most of the MAEs of the atoms were within 0.02e as well, except for *Fe*^2+^, which reached a maximum of 0.047e. Moreover, the Pearson correlation coefficient (*r*) between the predicted charges and the ESP charges of the RFR model in all three states was above 0.96. These data demonstrated that the model had high prediction accuracy, especially for N1 and N2. At the same time, the MAE and error standard deviation were close in the three states, indicating that our RFR model had good stability.

**Table 2:** The performance of prediction using RFR model with symmetric functions for three spin states.

**Atoms**	**Predicted values (e)**			**MAE**			**Error std**.			**Pearson coefficient**		
	**Singlet**	**Triplet**	**Quintuplet**	**Singlet**	**Triplet**	**Quintuplet**	**Singlet**	**Triplet**	**Quintuplet**	**Singlet**	**Triplet**	**Quintuplet**
*Fe*^2+^	1.390	1.382	1.459	0.048	0.046	0.047	0.051	0.049	0.050	0.978	0.980	0.982
N1	−0.390	−0.382	−0.390	0.013	0.014	0.013	0.014	0.014	0.014	0.991	0.991	0.991
N2	−0.387	−0.378	−0.385	0.014	0.014	0.014	0.014	0.014	0.014	0.991	0.990	0.991
N3	−0.457	−0.449	−0.458	0.013	0.013	0.012	0.014	0.014	0.014	0.988	0.988	0.989
N4	−0.451	−0.443	−0.452	0.014	0.014	0.014	0.015	0.015	0.015	0.986	0.986	0.986
C5	0.350	0.357	0.374	0.015	0.015	0.015	0.016	0.016	0.016	0.985	0.984	0.984
C6	−0.369	−0.371	−0.377	0.018	0.018	0.018	0.019	0.019	0.019	0.973	0.972	0.972
C7	0.363	0.368	0.384	0.016	0.016	0.016	0.017	0.018	0.017	0.978	0.977	0.977
C8	0.371	0.375	0.390	0.015	0.016	0.015	0.016	0.017	0.016	0.978	0.977	0.978
C9	−0.372	−0.373	−0.378	0.018	0.018	0.019	0.019	0.019	0.020	0.971	0.970	0.967
C10	0.348	0.354	0.370	0.016	0.016	0.015	0.017	0.017	0.016	0.983	0.983	0.984
N11	0.052	0.051	0.007	0.004	0.005	0.006	0.005	0.005	0.008	0.988	0.985	0.971
O12	−0.217	−0.266	−0.306	0.005	0.007	0.008	0.006	0.009	0.012	0.990	0.987	0.987
O13	−0.229	−0.224	−0.238	0.002	0.003	0.004	0.004	0.006	0.006	0.987	0.970	0.978
Mean				0.015	0.015	0.015	0.016	0.017	0.017	0.983	0.981	0.981

**Figure 5:**
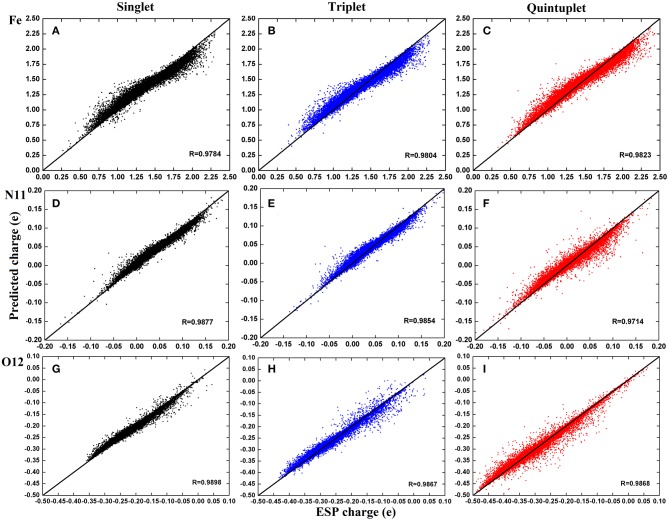
Distribution of predicted charge of atoms *Fe*^2+^, N11, and O12 in three spin states. The illustration use **(A–C)** for *Fe*^2+^; **(D–F)** for N11; **(G–I)**. The color codes are Black for singlet state, Blue for triplet state, and Red for quintuplet state.

For clarity, we further selected three atoms—*Fe*^2+^, N11, and O12—to plot their charge distributions for comparison (Figure 5). As shown in Figure 5, the predicted charges of the RFR model are basically gathered around the straight line *y* = *x*; they were very close to the high-precision charges calculated by DFT, indicating that our model achieved satisfactory accuracy.

By carefully comparing the distribution of each atom in different spin states, it can be found that the predicted values of *Fe*^2+^ have a good aggregation and few scattered points. However, the predictions are larger when the corresponding ESP charges are <1.5e and smaller when they are above 1.5e. The aggregation centers in the three states were different but essentially distributed in 0.5–2.25e, which is consistent with the analysis in Figure 5. For N11 atom, the predictions were more concentrated in the singlet and triplet, as there existed a few scattered points in the quintuplet. For O12 atoms, the correlation coefficients in all three spin states exceeded 0.98, and although there were some scattered points, the distribution was uniform. Finally, by comparing the distribution of different atoms in the same spin state, it can be found that the predictions in the triplet state were more concentrated overall. In summary, it was demonstrated that our model can predict the atomic charge of most structures well.

### 3.3. Charge Prediction of RFR Model With Manually Selected Structural Parameters

**Figure 6:**
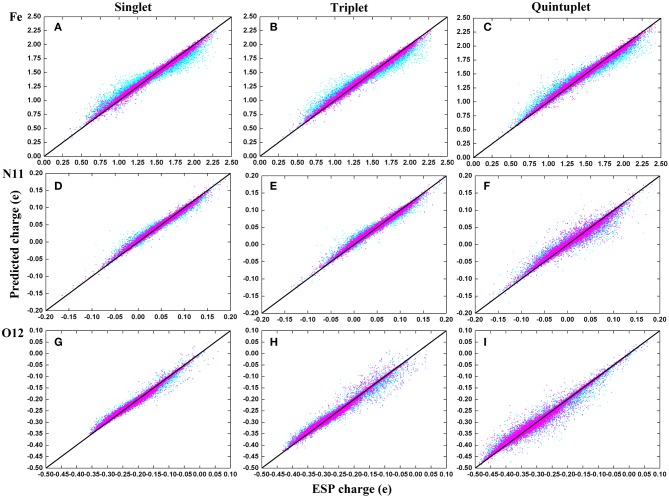
Comparison on the performance of two descriptors of RFR predictions in three spins states. The illustration use **(A–C)** for *Fe*^2+^; **(D–F)** for N11; **(G–I)**. Cyan corresponds to the RFR with a symmetric function, and magenta represents the RFR with 11 structural parameters.

**Figure 7:**
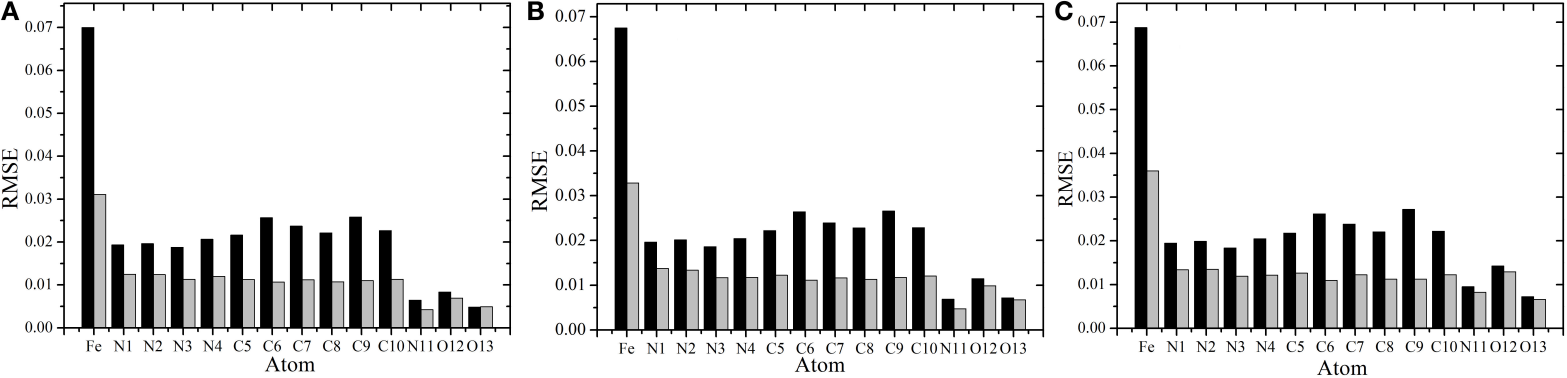
Comparison of the root mean square error(RMSE) using different RFR models. **(A–C)** Represent the RMSE of each atom in singlet, triplet, and quintuplet state, respectively. The black bar is the RFR model using symmetric functions, and the gray bar is the RFR model with 11 manual selected structural parameters.

To compare the performance of the RFR models with different descriptors, we manually screened 11 parameters to describe the molecular structure, including eight bond lengths (Fe-N1,Fe-N2, Fe-N3, Fe-N4, Fe-N11, Fe-O12, Fe-O13, and O12-O13), one bond angle (Fe-O12-O13), and two dihedrals angles (N2-Fe-N1-C10 and N1-Fe-N2-C5). The same process and method were used for RFR model training and prediction. A comparison of the prediction performance of selected atoms (*Fe*^2+^, N11, and O12) is shown in Figure 6, and, for comparison, the root mean square error RMSE of the prediction of each atom in different spin states was further calculated and is shown in Figure 7.

It can be seen clearly from Figure 6 that both models have good prediction performances, and the same model has a similar RMSE of predictions for different spin states. When 11 structural parameters were used as descriptors, however, the prediction values were more concentrated, and the model prediction performance was better than with in the case of symmetry functions. The average RMSE and the RMSE of each atom were reduced. Among these, the RMSE of *Fe*^2+^ reduced from 0.07 to 0.0035e, which is a maximum 0.06e improvement. We think that this is partially due to the use of a dihedral angle as the descriptor, which is a four-body term and is not included in the symmetry function.

In conclusion, choosing different descriptors will affect the prediction performance of the RFR model; the 11 manually selected parameters can better describe the molecular structure and thus achieved better results. At the same time, however, it should be noted that the difference between the two cases is not significant. As shown in Figure 7, the RMSE of *Fe*^2+^ is relatively larger, but its fluctuations are still below 0.04e, and the variations of RMSE for other atoms are all below 0.02e. This indicates that, even if there is no empirical experience involved, the RFR model with symmetry functions can achieve satisfactory predictions, and the advantage is that it can be automatized.

## 4. Conclusions

This study aimed at exploring the spin crossover phenomenon in the model heme system according to the characteristics of atomic charge distribution in different spin states with conformation. The random forest method was introduced to construct a prediction model of multi-spin variable charge, which can provide a separate prediction for a single atom.

In this model, symmetry functions were used as descriptors to describe the atomic chemical environment. The model was trained in conjunction with the ESP charges to predict the atomic charge in different spin states. Meanwhile, in order to compare the prediction performance, 11 artificially selected structural parameters were also used as the input of RFR model. The results showed that, when the 11 selected parameters were adopted, the prediction was more accurate, but it was not suitable for automation considering the involvement of human experience. In contrast, the RFR model using symmetry functions can achieve a good trade-off between calculation accuracy and efficiency, realize automatic processing, and provide separate prediction for a single atom. It should be noted that, in this method, the transformation of coordinates is a time-consuming pre-processing process, but it avoids the problem of inconsistent calculation of energy or force in Cartesian coordinates. When the number of descriptors is large enough, the random forest algorithm is very effective. This study is only a preliminary exploration of the heme force field, and there are still many deficiencies. In future work, we will further improve the calculation method of the multi-spin state variable charge force field parameters.

## Data Availability Statement

The datasets generated for this study are available on request to the corresponding author.

## Author Contributions

JG: conceptualization. QL, WZ, X-HH, and L-HB: methodology. WZ and L-HB: validation. WZ, L-HB, and JG: writing–original draft, writing–review, and editing. JG: project administration and funding acquisition.

### Conflict of Interest

The authors declare that the research was conducted in the absence of any commercial or financial relationships that could be construed as a potential conflict of interest.
